# Hierarchical levels of organization of the Brazil nut mesocarp

**DOI:** 10.1038/s41598-020-62245-y

**Published:** 2020-04-22

**Authors:** Marilia Sonego, Claudia Fleck, Luiz Antonio Pessan

**Affiliations:** 1 Department of Materials Engineering, Federal University of São Carlos, via Washington Luiz, Km 235, 13565-905 São Carlos, SP Brazil; 2 Graduate Program in Materials Science and Engineering (PPGCEM), Federal University of São Carlos (UFSCar), via Washington Luiz, Km 235, 13565-905 São Carlos, SP Brazil; 3 Materials Science and Engineering, Technische Universität Berlin, Berlin, 10623 Germany

**Keywords:** Biological physics, Plant biotechnology, Plant cell biology

## Abstract

Aiming to understand Nature´s strategies that inspire new composite materials, the hierarchical levels of organization of the Brazil nut (*Bertholletia excelsa*) mesocarp were investigated. Optical microscopy, scanning electron microscopy (SEM), microtomography (MicroCT) and small-angle X-ray scattering (SAXS) were used to deeply describe the cellular and fibrillary levels of organization. The mesocarp is the middle layer of the fruit which has developed several strategies to avoid its opening and protect its seed. Fibers have a different orientation in the three layers of the mesocarp, what reduces the anisotropy of the structure. Sclereids cells with thick cell walls fill the spaces between the fibers resembling a foam-filled structural composite. The mesocarp has several tubular channels and fractured surfaces which may work as sites for crack trapping and increase toughness. The thick and lignified cell wall of sclereids and fibers and the weak interface between cells can promote a longer and tortuous intercellular crack path. Additionally, fibers with high strength and stiffness due to microfibrils oriented along the main cell axis (µ = 0° to 17°) were identified in the innermost layer of the mesocarp. Such an understanding of each hierarchical level can inspire the development of new cellular composites with improved mechanical behavior

## Introduction

The mesocarp layer of *Bertholletia excelsa* fruit, the seed of which is known as brazil nut, is an impressive structure. It is a shell capable to resist falls as high as 50 m and compression forces higher than 10 kN^1^. Such a strong natural structure has great potential as a source for bioinspiration to produce new high performance composites^1,2^. The impressive properties of the mesocarp arise from its hierarchical structure, which emerged from millions of years of evolution.

This hierarchy is the main factor to explain how relatively weak components organized in a complex way can result in a system with outstanding properties^3^. Four hierarchical levels of structuring have been described for plants: macroscopic, cellular, fibrillar, and molecular^3^. Each hierarchical level has its own contribution to the overall properties of the natural composite.

On the largest length-scale, the “macroscopic” level, trunk, leaves, roots, flowers, and fruit are distinguished, and it is analyzed how the association of different tissues leads to different geometries and functions in the plant. At this level, the mesocarp is described as a spherical or elliptical shell of 10–12 cm diameter with a wall thickness of approximately 1 cm. Its function is to protect the seeds against predators and impact on the ground when the fruit falls from the tree^1,4,5^. The mesocarp has a rough surface with a peduncle and an opercular opening on opposite sides of the fruit. The opening is a hole with a diameter of approximately 2 cm, which is, however, not large enough to allow the dispersion of the seeds, as it was millions of years ago.

On the cellular hierarchical level, the cells and vegetable tissues are identified together with possible preferential cell orientations. The main vegetable tissues are parenchyma, collenchyma, sclerenchyma, and vascular system; within these, cells are classified regarding their morphology, wall thickness, composition, and function.

The fibrillar hierarchical level comprises the cell walls of the vegetable cells. This structure can be described as a composite of cellulosic microfibrils reinforcing a matrix composed of lignin, hemicellulose and other substances. The microfibrils with diameters spanning 10–20 nm are formed by an organized aggregation of approximately 36 cellulosic molecules^3,6^. The fibrils are the main structural component of the cell wall. The orientation of the microfibrils defines the three layers found in a plant cell walls, the middle lamella, the primary and the secondary wall. The middle lamella is rich in lignin and it forms the connecting adhesive interface between different cells. The primary wall is thin and composed mainly of hemicelluloses, pectin, and structural proteins with randomly oriented cellulosic microfibrils^3,7^. The secondary wall has three layers, named S1, S2, and S3. Due to the parallel alignment of microfibrils in a matrix of lignin and hemicellulose this layer gives the single cells their mechanical stability. The cellulosic microfibrils have a helical arrangement with respect to the long axis of the cell. The helical, or “microfibril” angle (µ) has a great influence specifically on the mechanical response of fiber cells^8,9^.

The molecular level comprises the basic components of a vegetable cell, which are cellulose, lignin, and hemicellulose. It was shown previously that the mesocarp of Brazil nuts contains 15.9% of α-cellulose, 15.7% of hemicellulose and 58.2% of lignin among other substances. This is a relatively high lignin content, even compared to other nutshells^1,10^.

This work reports on observations of the hierarchical structure and organization on the cellular and fibrillar level of the mesocarp layer of *Bertholletia excelsa*. Based on the structural analysis several possible explanations for the outstanding impact and puncture resistance of the *Bertholletia excelsa* mesocarp were suggested, as well as options for biomimetic transfer strategies.

## Results

### Cellular hierarchical level

**Figure 1:**
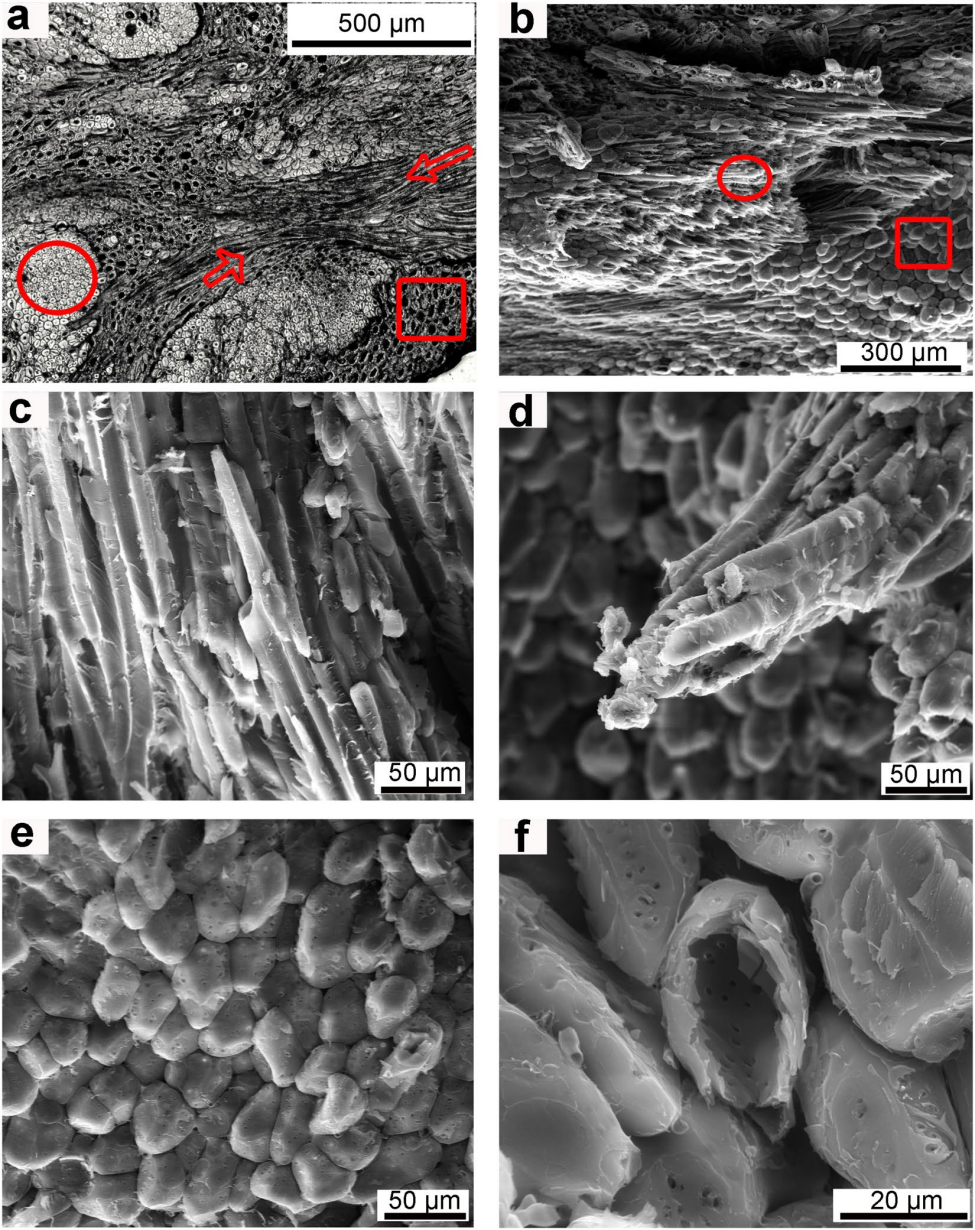
Cellular hierarchical level: (**a**) optical micrograph showing cross-sections of fibers (circle), longitudinal sections of fibers (arrows) and sclereid cells (square); SEM micrographs of fracture surfaces giving a general overview (**b**) of fibers (circle) and sclereids (square), (**c**) a bundle of fibers; (**d**) fractured fibers, (**e**) elliptical sclereid cells and (**f**) fractured sclereid cells with empty lumen.

**Figure 2:**
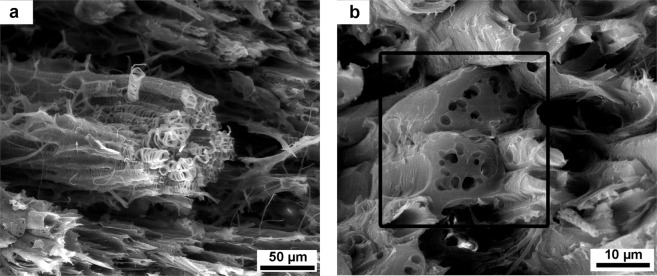
SEM micrographs of the vascular system of the mesocarp: (**a**) xylem is constituted by tracheid cells associated with bundles of fibers and (**b**) phloem is made of sieve elements which are highlighted by a black square.

The mesocarp of *Bertholletia excelsa* has two main constituents: the sclerenchyma tissue, made of fibers and sclereids cells (Fig. 1(a,b)), and the vascular system (Fig. 2).

Fibers are elongated cells, usually organized as bundles (Fig. 1(a,c,d)), which are dead and hollow at maturity due to their high lignin content^11^. The fiber cells of the mesocarp have a diameter of approximately 20 µm and a cell wall thickness of 5–10 µm.

Sclereid cells (Fig. 1(a,b)) have an isometric or elliptical shape, with a diameter of approximately 30 µm, a thick and lignified cell wall about 5 µm thick and an empty lumen, as can be seen in Fig. 1(e,f).

Comparing fibers (circle) and sclereids (squares) in Fig. 1(a), the cell diameter/wall thickness ratio seems smaller for sclereids than for fibers. This impression proves true when the ratio is calculated, giving approximate values of 56% for sclereids and 75% for fibers

The vascular system of the mesocarp is composed of xylem (Fig. 2(a)) and phloem (Fig. 2(b)).

Xylem is formed by tracheid cells, as shown in Fig. 2(a). Its function is to transport water through the mesocarp. The tracheid cells are usually surrounded by fibers, that provide mechanical stability to the structure. This kind of cell is common among other nutshells, like in macadamia and coconut^12,13^. Phloem cells are responsible for transporting nutrients through the plant and are formed by sieve elements, as shown in Fig. 2(b).

**Figure 3:**
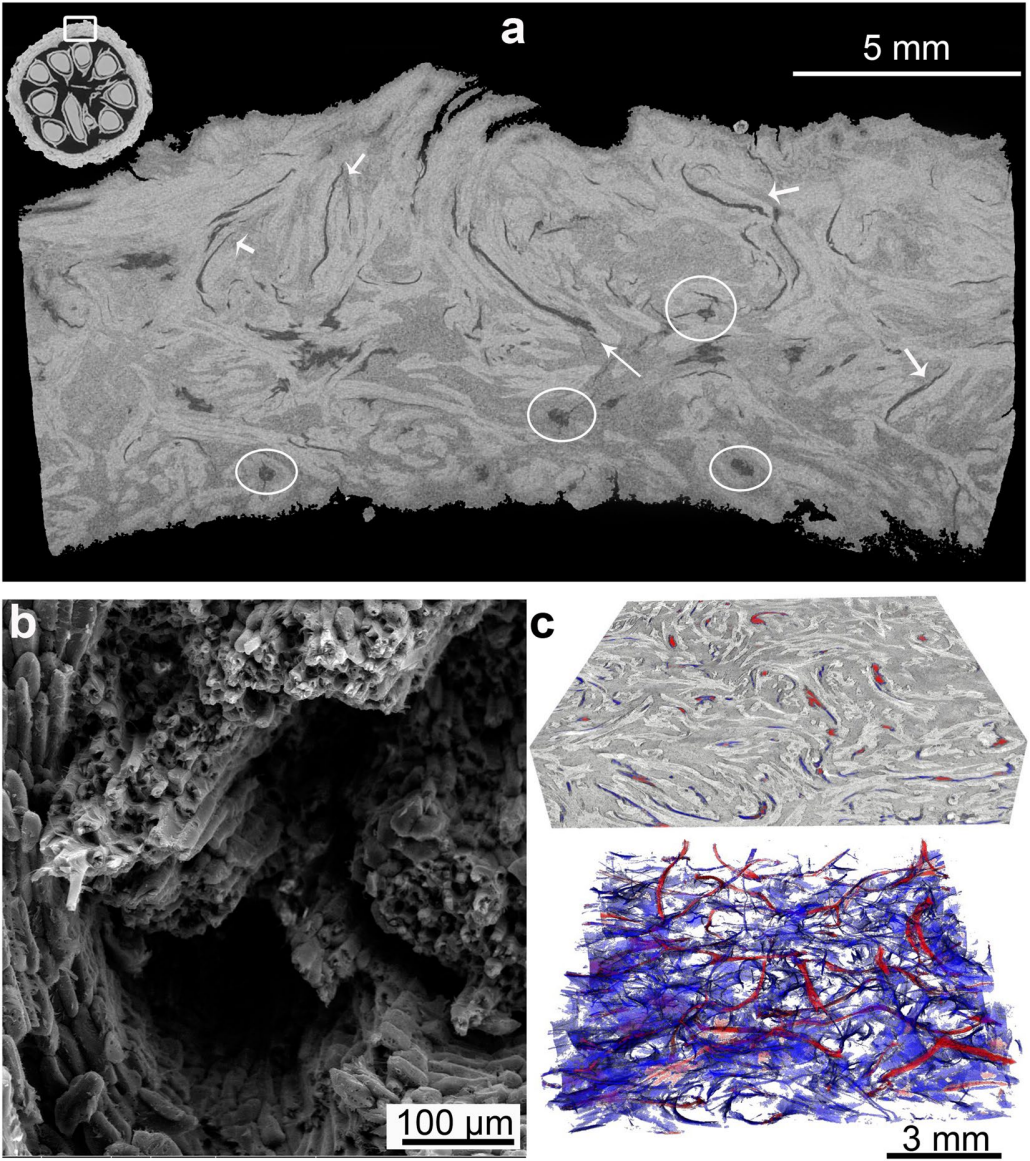
Voids in the mesocarp: (**a**) microCT slice (2D) of a mesocarp piece (latitudinal view) where circles and arrows highlight the channels and cracked surfaces, respectively; (**b**) SEM image showing channel surrounded by fiber cells; (**c**) 3D model of channels from microCT analysis where channels are in red and cracked surfaces are in blue.

Another striking observation is several natural voids found throughout the mesocarp structure, as shown in Fig. 3.

These voids can be vascular channels (marked by circles in Fig. 3(a)) or they resemble cracks (marked by arrows in Fig. 3(a)). Figure 3(b) shows an SEM image of a channel while Fig. 3(c) shows a 3D model of a volume of interest cropped from microCT data. There, the cracked surfaces are in blue and the channels are in red. Additionally, some secondary cracks can nucleate from the channels as shown in the circled regions of Fig. 3(a). The amount of channels and cracked surfaces, estimated from the porosity of one mesocarp with no previous treatment, is 13% ± 2.

**Figure 4:**
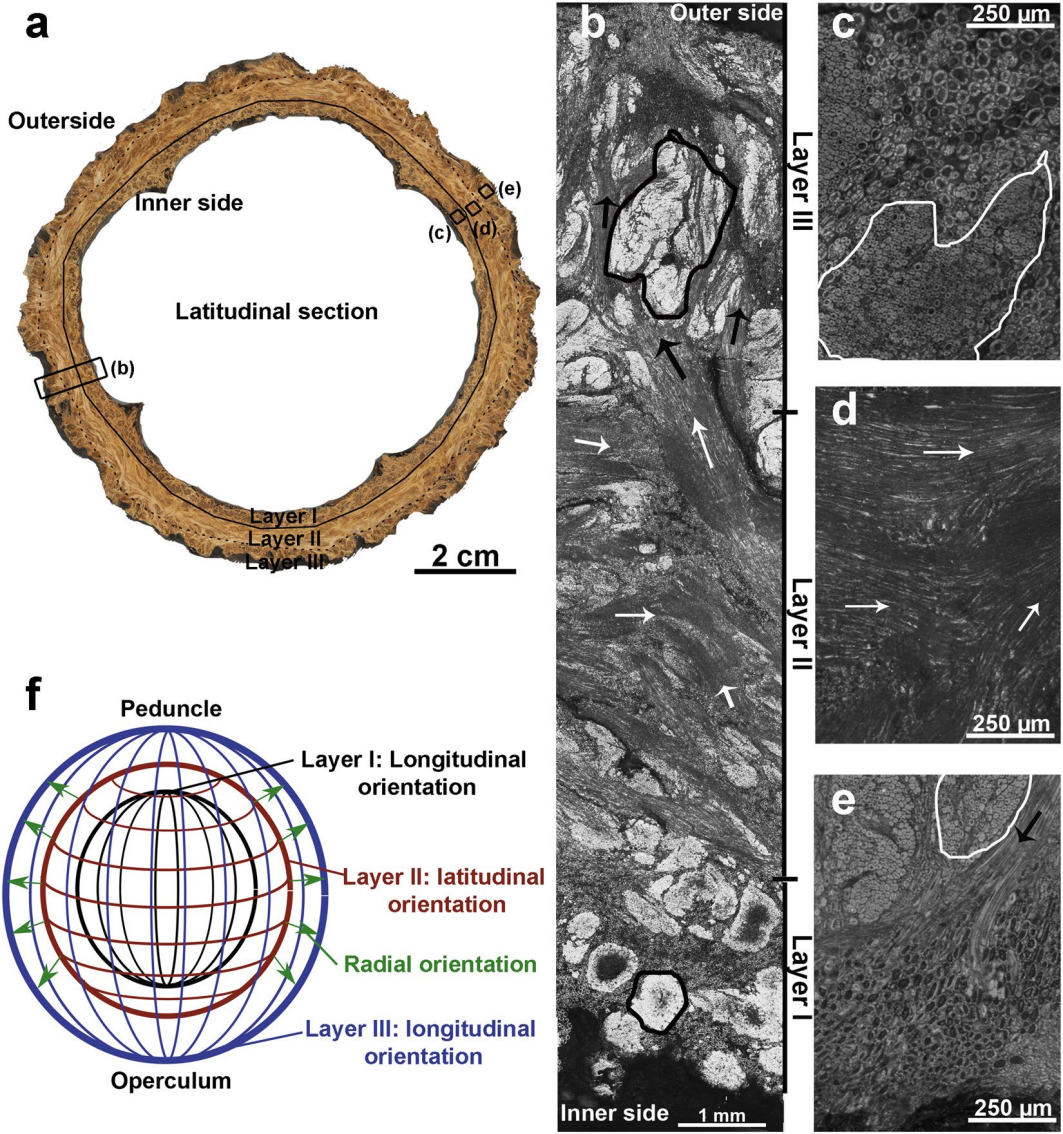
Preferential orientation of fibers in the mesocarp: (**a**) digital macrograph of a latitudinal section of the mesocarp with three visible layers (I, II, III), black rectangles indicate the regions visualized with optical microscopy in the following pictures; (**b**) optical micrograph of complete thickness of the mesocarp where the three layers are indicated; optical micrograph from (**c**) layer I; (**d**) layer II; (**e**) layer III, where the cross-section of bundles of fibers are countered with lines, longitudinal sections of bundle of fibers are highlighted with arrows (black for radial orientation and white for latitudinal orientation) and (**f**) schematic drawing that shows the preferential orientation of fibers in each layer.

The fiber bundles are frequently entangled, resembling a weaved organization. Nevertheless, in certain areas, a preferential fiber bundle orientation is observed, as shown in Fig. 4. The fiber cells are often found together with sclereid cells filling the spaces between the bundles (see Figs. 1(a) and 4(b)), or they are associated with the xylem cells. Based on fiber orientation, three different layers can be distinguished in the mesocarp, as shown in the macroscopic view of a latitudinal section in Fig. 4(a) and the higher magnified views in Fig. 4(b).

The innermost layer, delineated with a solid line in Fig. 4(a) and designated as “layer I” in the following, exhibits a preferential orientation of the fiber bundles in the longitudinal direction, running from the peduncle to the opercular opening. In the light microscope, (Fig. 4(b)) bright circular areas are observed. These are cross-sections of fiber bundles, as can be better seen at higher magnification (Fig. 4(c)). The fiber bundles are surrounded by sclereids cells with bigger cross-sections.

“Layer II”, between the solid and the dotted lines in Fig. 4(a), has a higher amount of fibers with a preferential latitudinal orientation. It is characterized by dark regions with elongated shape, which are longitudinal sections of fiber bundles, as shown in Fig. 4(b,d). The most external layer, “layer III”, delineated with a dotted line in Fig. 4(a), hardly shows any preferential fiber orientation. Only some fiber bundles with a longitudinal orientation from the peduncle to the opercular opening, similar to the orientation in layer I, can be found. Thus, layers I and III have the same bright regions (marked by solid lines), identified as cross-sections of the bundles (Fig. 4(b)). Additionally, layer III also contains bundles of fibers with a radial orientation and running from layer II to layer III and throughout the latter (Fig. 4(b,e)). Figure 4(f) shows a schematic drawing summarizing our observations of the preferential fiber bundle orientation in the different layers.

**Figure 5:**
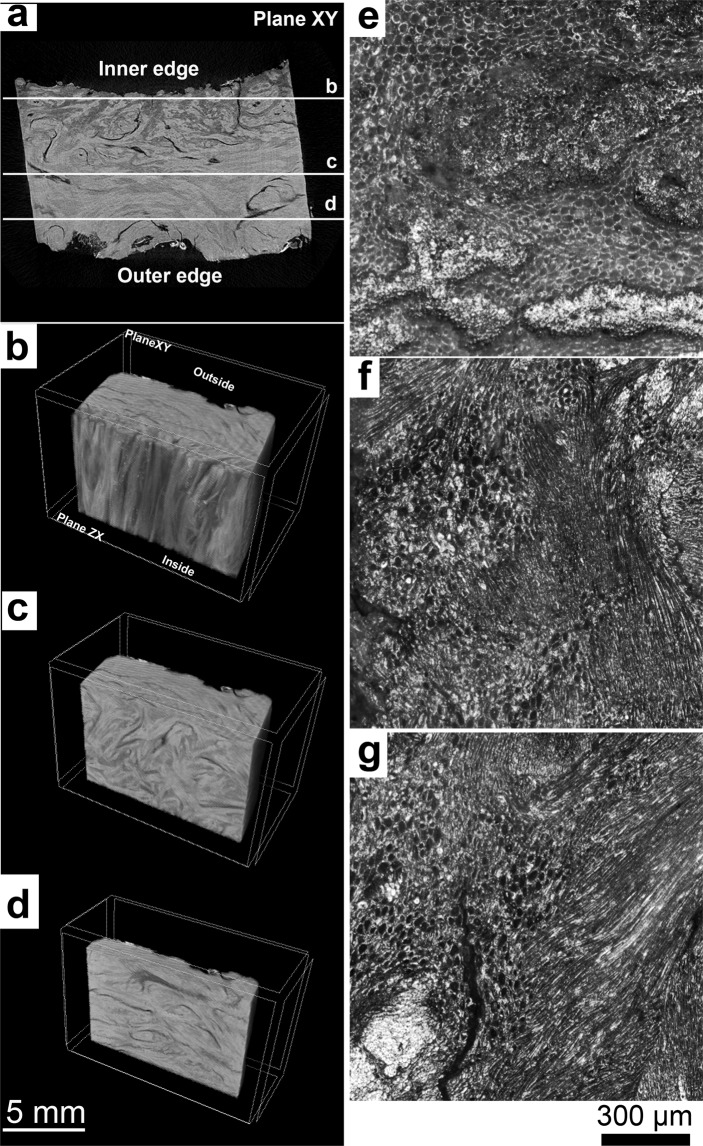
Density gradient in the mesocarp structure: (**a**) microCT slice (2D) of mesocarp piece where thickness sections in a latitudinal view are indicated in the microCT 3D model as (**b**) layer I; (**c**) layer II and (**d**) layer III. Optical micrograph showing: (**e**) layer I; (**f**) layer II and (**g**) layer III from a latitudinal section of the mesocarp.

Assuming that the mesocarp has a homogneous elemental composition, the grayscale contrast of microCT images is caused by a density gradient. As shown in Fig. 5(a), the mesocarp of Brazil nut has a density gradient from inside to outside. In layer, I (Fig. 5(a)) – near the inner edge, has darker regions that represent a less dense material. Additionally, a cut in layer I (Fig. 5(b)) clearly shows the oriented fiber bundles, which are denser than sclereids cells. However, the density contrast progressively deteriorates, assuming lighter shades of gray, as we reach layers II and III. The cells present in each layer are shown in Fig. 5(e–g). No significant difference in cell size, geometry or wall thickness can be distinguished among cells in layers I (Fig. 5(e)), II (Fig. 5(f)) and III (Fig. 5(g)).

### Fibrillary hierarchical level

**Figure 6:**
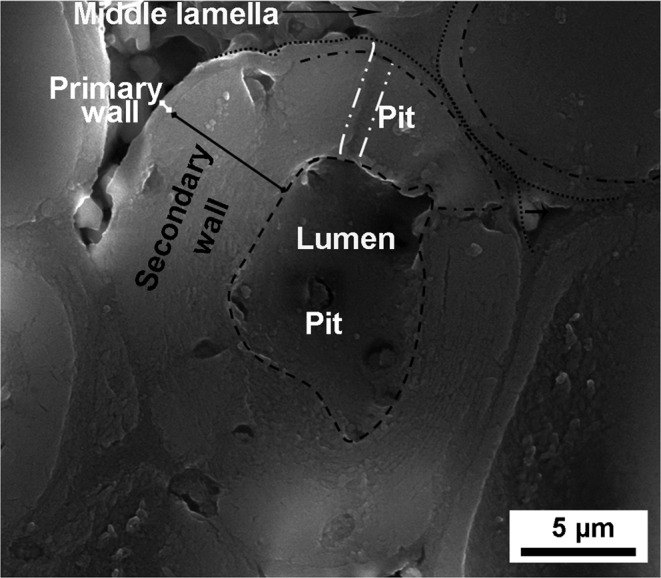
SEM image of polished mesocarp pieces showing the cell wall of sclereid cell where middle lamella, primary, secondary wall, and pits are highlighted.

The layers of the cell wall of the sclereid cell are shown in Fig. 6. The primary wall is much thinner than the secondary one, which represents most part of the cell wall^14^. The middle lamella joins neighboring cells, constituting a weak interface between cells with holes and empty spaces. The pits, channels connecting cells, are also highlighted in Fig. 6.

**Figure 7:**
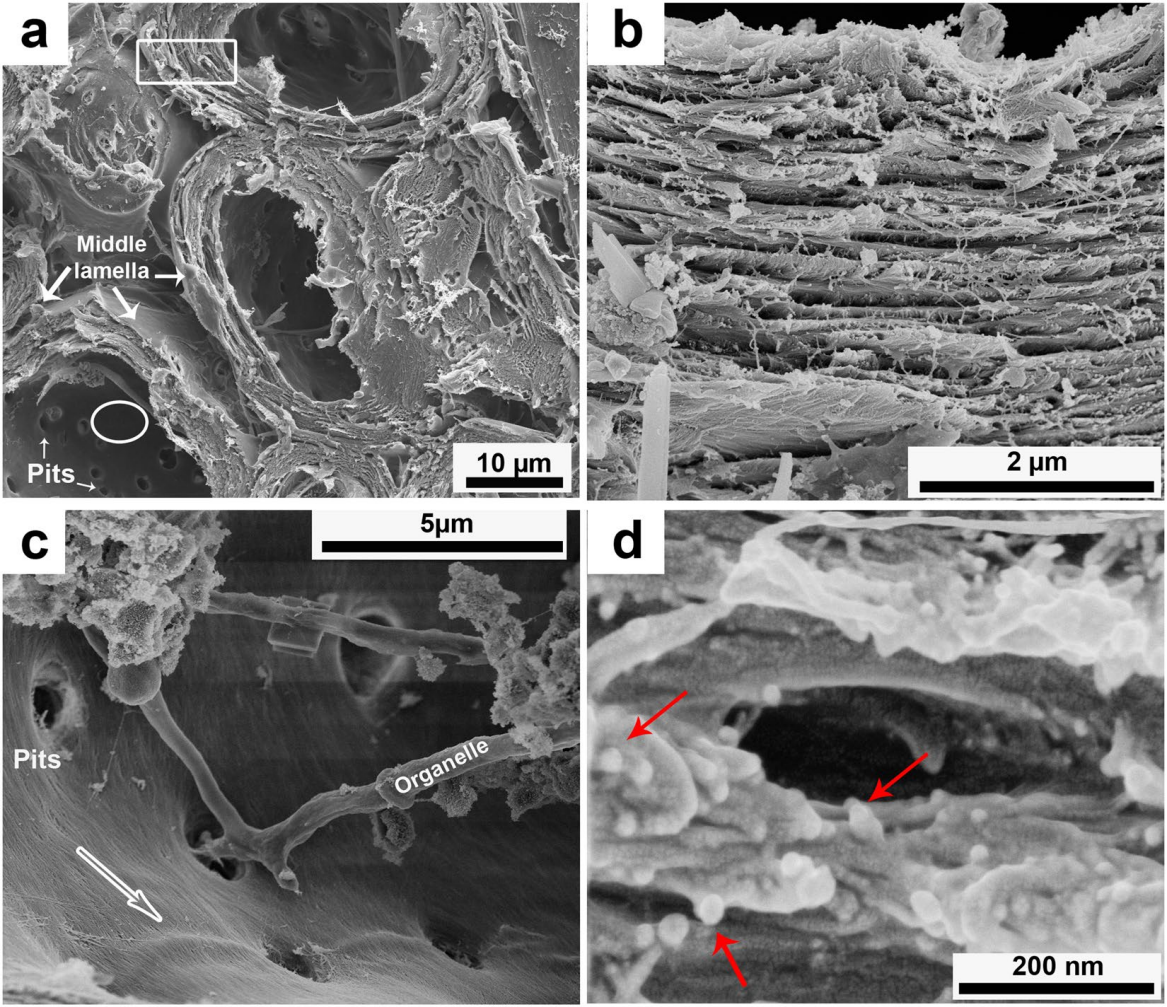
SEM images of sclereid cell wall: (**a**) general view of a sclereids cell walls showing middle lamella (white arrows); primary and secondary wall (white rectangle), lumen-wall or S3 layer (white circle) and pits (white arrows) (**b**) layers of a cell wall; (**c**) interior of sclereids cell showing its pits, microfibril orientation (white arrow) in a S3 layer of secondary wall and a remaining cellular organelle; (**d**) high resolution image of layers of S2, where red arrows indicate the cross-section of microfibrils.

SEM images of a fractured sclereid cell wall are shown in Fig. 7. The sclereid cells have an elliptical shape (Fig. 7(a)). The thick cell wall is formed by layers of oriented microfibrils in a lignin-hemicellulose matrix (Fig. 7(b)). Note the weak middle lamellae indicated by black arrows in Fig. 7(a). In Fig. 7(b), it is clear that the cell wall, and especially the thickest S2 layer, is made of several sublayers. Figure 7(c) shows the S3 layer of the secondary wall, also called the lumen layer. There, one can see the microfibril orientation of the S3 layer, indicated by a white arrow. In this image, there are several pits and an organelle that remained after cell death. Finally, Fig. 7(d) shows the S2 layer in higher magnification, where the cellulosic microfibrils are visible (highlighted by arrows).

**Figure 8:**
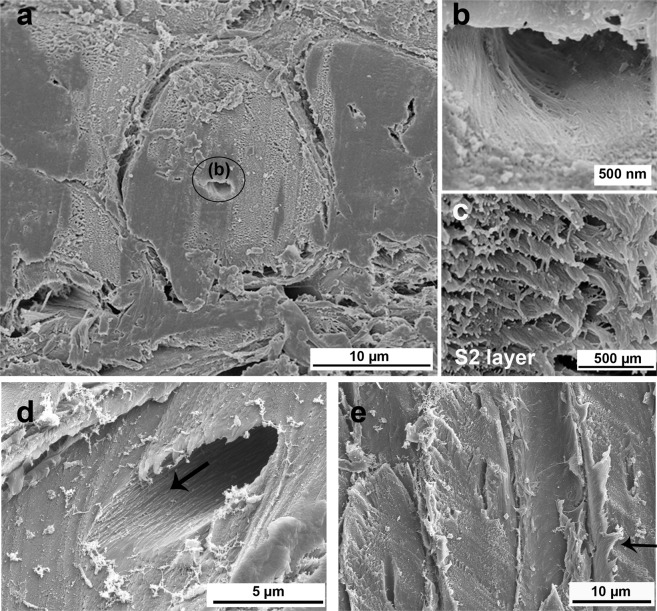
SEM images of fiber cell wall: (**a**) general view of a fiber cross-section; (**b**) empty lumen showing the S3 layer of secondary wall; (**c**) oriented microfibrils and (**d**) low microfibril angle in fiber lumen indicated by arrow and (**e**) longitudinal sections of fibers where arrow highlight plastic deformation of one fiber layer.

Figure 8(a) shows a cross-section of a fiber. The S3 layer (circled region) is visible in the center and can be seen in detail at higher magnification (Fig. 8(b)). The S3 layer is very thin and has oriented microfibrils. The major part of the fiber cell wall consists of the S2 layer (Fig. 8(c,d)). The microfibrils in the S2 layer (Fig. 8(c)) have a helical orientation along the longitudinal axis of the fiber cell with a low angulation (indicated by an arrow in Fig. 8(d)). The S2 layer is responsible for the cell mechanical support due to its high thickness and helical microfibril orientation. Figure 8(e) shows an outer layer which in contrast to S2, is capable of great plastic deformation.

**Figure 9:**
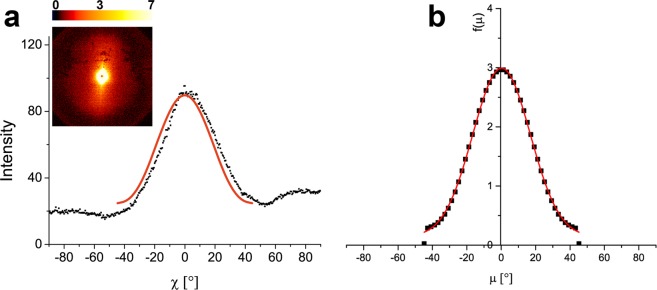
Microfibril angle of fibers: (**a**) small-angle scattering pattern obtained from one fiber bundle from layer I of the mesocarp, where just one single streak is visible and integration over q leads to a single peak (**b**) MFA distribution f(µ) with a peak in 0° and a Gaussian width of 17°.

Depending on the microfibril angle (µ), the fiber cell can have different stiffness and ductility. Different fibers may have different µ, therefore the microfibril angle distribution, f(µ), obtained by small-angle X-ray scattering (SAXS) is a reliable measurement. The SAXS patterns of several fibers in one bundle from the mesocarp layer I has only one streak which after integration over q, resulted in one single peak (Fig. 9(a)). The f(µ), shown in Fig. 9(b), indicates a mean microfibril angle at µ = 0° and distribution width of 17°.

## Discussion

**Figure 10:**
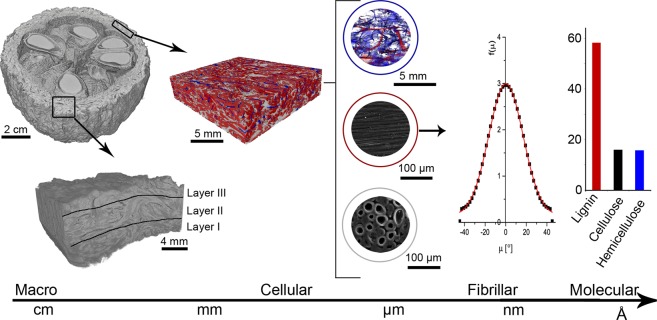
Hierarchical levels of organization of the mesocarp of *Bertholletia excelsa* fruit.

Figure 10 shows the hierarchical organization of the mesocarp. Considering the macroscopic level, the spherical or elliptical form, besides helping the fruit dispersion, has no suture to facilitate its opening, as observed in other fruits and nuts, such as e.g. macadamia nutshell^15^. The opercular opening, once large enough to allow seed dispersion, has evolved to hinder access to the seeds. Nevertheless, the peduncle and the opercular opening must inevitably be weak points in the shell. The fruit is only opened, allowing the seed germination, with the help of a rodent disperser, the agouti^1,4^.

On the cellular level, the presence of hollow and empty cells is a well-known strategy of Nature to simultaneously optimize stiffness, strength and overall weight^16^. However, the high volume ratio of channels and cracked surfaces is intriguing. The channels can be formed by the death and absorption of vascular cells. As the mesocarp only has dead cells after maturation, the transport of nutrients is useless for the mature fruit, therefore the phloem cells can be absorbed by mesocarp leaving hollow channels behind. Similar tubular channels have also been observed by Gludovatz *et al*.^13^ when studying the mechanical properties of coconut endocarp. They pointed out that these channels can work as sites for crack trapping and deflection in coconut endocarp, thus contributing to an improved toughness during crack growth. Fleck *et al*.^15^ found a channel-like structure in macadamia, which they identified as vascular bundles. During tensile loading of notched specimens, the channels deflected the main crack, working as a toughening mechanism; however, considering their stress-raising factor of 2 to 3, they can also lower the strength and resistance of the nutshell. Therefore, whether the channels – having once, during fruit development, an important nutrition function – are indeed toughening elements, that have evolved during evolution, or whether they are just there because they cannot be removed during maturation and just have, by chance, the positive toughening effect, is an interesting question. However, it is beyond the scope of this paper and should be addressed in greater detail in future work.

The cracked surfaces may have the same dual effect as the channels: on one hand, they may well work as toughening mechanisms by trapping and deflecting advancing cracks, while on the other hand, they surely decrease the elastic modulus of the mesocarp and act as stress-raisers. They may be formed due to hydrothermal deformations in the weak interface of a bundle of fibers and sclereid cells, as several of them follow the orientation of the fiber bundles (light grey in Fig. 3(a)). Whether these fractured surfaces have a toughening effect or whether they are just purely damaging to the mechanical resistance of the shell can only be identified by a detailed analysis of crack growth and fracture. As well-known for microcracks in bone^17,18^, dentin^19^ and ceramic materials^20^ the beneficial effect of these voids to the mesocarp toughness will strongly depend on void size, shape, orientation, and spatial distribution^18,21^. Therefore, the mimicking of such voids in a bioinspired material requires a deeper understanding of how and why these voids form, how they are distributed, and how they interact with mechanically induced cracks during mechanical loading of the shell.

Several nutshells are composed of fibers and sclereids^12,13,22–24^. However, an interesting feature in the mesocarp of Brazil nut is how the sclereids are arranged, filling the gaps between the fiber bundles^1^. This arrangement is uncommon in other nutshells such as macadamia^12^, coconut^25^ and babassu^22^, where fibers and sclereids are present in different layers of the structure. The cell arrangement in the Brazil nut mesocarp reminds of a foam-filled sandwich structure, as used in the aerospace industry where there is a skin made of a stiff material and the core is filled with foam^16^. The foam core reduces the density of the composite while improving its bending stiffness^26^.

The preferential fiber bundle orientation in the longitudinal and the latitudinal directions in different layers is a way to decrease anisotropy of properties caused by fiber reinforcement. Additionally, the boundaries between layers may deflect propagating cracks. As shown in Fig. 4(b), the fiber bundles are partially disoriented in one of the layers (layer III), even though a preferential orientation still prevails. As we may safely assume that cracks will propagate preferentially along the weak fiber interfaces, such partial disorientation can increase the crack path length which would then be beneficial to the mesocarp toughness. Another advantage of the layered mesocarp structure is the difference of hardness between different layers^1^. As shown in Fig. 5, the mesocarp has a density gradient which may explain the difference in hardness among the Brazil nut mesocarp layers. Such a density gradient must be caused by the relative amount of fibers and their orientation. In layer I, fewer fiber bundles are seen and they have a more pronounced orientation. In layers II and III, fiber bundles are increasingly entangled, reducing the spaces filled with sclereid cells, which can explain the low-density contrast in the microCT images. Structural gradients in plant structures are a known natural strategy to yield property gradients (e.g. in stiffness), and they improve the cohesion of tissues with different properties and avoid stress concentrations^27^. For example, the endocarp of the cocoyol palm tree (*Acrocomia mexicana*)^24^, has a harder external layer to protect seeds against predators trying to penetrate the shell and an inner layer with mechanisms to enhance shell toughness. Nature employs a variety of strategies to achieve such properties gradients, like adjusting the degree of lignification, as in the stem of *Washingtonia robusta*^28^, differential cell wall thickening, as in *Phyllostachys pubescens*^29^, changes in cell geometry, as seen in the endocarp of the cocoyol^24^, or by introducing density gradients by different pore sizes, as in *Citrus maxima*^30,31^. Whether other mechanisms are involved in the mesocarp density gradient, specifically different degrees of lignification, have to be investigated in the future.

On the fibrillar level, the cell wall is a complex structure with several inspiring structural strategies of which the helical orientation of the microfibril in the S2 layer is an important one. The microfibril angle (µ) affects the mechanical behavior of the single fibers and, therefore, influences the kind of reinforcement present in the mesocarp. According to Reiterer *et al*.^32^, an angle µ = 5° leads to fibers with high strength and stiffness while fibers with µ = 50° behave in a much softer way due to buckling effects. They further concluded that a balanced combination of strength, stiffness, and deformability and thus optimized toughness, is achieved with an intermediate angle of µ = 27°. According to SAXS results, the mesocarp fibers from one bundle in layer I are preferentially oriented along the main axis with an average angle of µ = 0° and a microfibril angle distribution (f(µ)) width of 17°. Therefore, the mesocarp has fibers with high strength and stiffness in layer I. Unfortunately, it was not possible to isolate fibers from layers II and III due to its entanglement, so the µ of most fibers in the mesocarp is still unknown and should be investigated in the future.

Although the S2 layer of the secondary cell wall is the main component responsible for the stiffness and ductility of the fiber, the primary wall also has an interesting mechanical behavior. According to Burgert and Keplinger^7^, the primary wall needs to be plastically deformable to adjust deformations during cell growth but it also needs a high degree of elasticity to allow the reversible movements of the cell. This interplay between plastic and elastic behavior has still not been understood. Figure 8(e) shows the mesocarp fibers with one layer with very plastic behavior which is an example of the complexity of a fiber cell.

Another interesting feature of the fibrillar level is that the thick cell wall of sclereid and fiber cells (Figs. 7 and 8) is very difficult to tear. As shown in Fig. 7, the cell wall of mesocarp sclereids have several layers of oriented microfibrils which is a potent obstacle to crack propagation because the cracks will preferentially advance through the (weaker) interfaces between the cells, preventing a straight crack path through the layer in orthogonal direction to its thickness, that is in the radial direction. When a crack follows the sclereids interfaces, it has a more tortuous and longer path and it may be even branched, resulting in high energy dissipation. The same effect is even more intense for fibers. As for sclereid cells, the interfaces between fiber cells are also an easier crack path than across the fiber, breaking it. Additionally, as the longitudinal axis of the fibers is larger than their latitudinal axis, the crack path is considerably longer and more tortuous than around the more equiaxed sclereid cells^33^.

Finally, on the smallest length scale, the mesocarp has a high lignin content consisting of a reticulum with short, linear, randomly crosslinked chains providing stiffness and strength, mainly under compression conditions^34^. This has been shown for coconut endocarp: the higher lignin content in an old and, therefore, more lignified coconut endocarp lead to a higher density and less porous cell walls, with higher strength and elastic modulus during tensile loading, and with a higher K_IC_^13^. Therefore, the high lignin content in the mesocarp may safely be assumed to improve its strength, stiffness and puncture resistance.

The description and understanding of the hierarchical level of the organization of mesocarp can inspire several strategies to improve the impact and puncture resistance of materials. Focusing on the macro and cellular levels, a bioinspired composite is proposed as follows.

Rigid closed-cell foams or syntactic foams – foams where the pores are hollow glass microspheres in a polymeric matrix - strongly resemble sclereids and are excellent model materials to mimic them. Syntactic foams or rigid foams usually uses a highly crosslinked resin (thermoset), that can have the same effect of the crosslinked lignin chains. Syntactic foams are better than rigid closed-cells foams for mimicking sclereids due to its strong cell walls made of glass and weaker interphase of polymeric matrix between the cells. This combination should promote intercellular versus intracellular crack propagation.

Compton and Lewis^35^, inspired by balsa wood, have produced lightweight cellular composites using 3D printing of fiber-reinforced filaments. These allow reproducing the orientation, distribution, and entanglement of fiber bundles by 3D printing of filaments containing glass or carbon fibers, for example. The printed material, containing oriented glass or carbon fibers, is similar to individual fiber cells that form a fiber bundle. Moreover, the printed structure should be specifically designed with free volumes, that may subsequently be filled with the foams mentioned above, similar to the way the sclereids fill the gaps around the fiber bundles.

The voids in the mesocarp structure can be reproduced by 3D printing of a teflon filament if this is desired. Teflon has poor adhesion to the surrounding material leading to very weak interphase, which may work in the same way as the voids do in the mesocarp. A similar strategy of using teflon to create defined voids that act as a notch has been adopted in fracture toughness tests of laminate materials^36,37^.

## Methods

### Materials

Brazil nut fruits were bought from Mais Castanhas Alimentos from Juina - MT and from a local producer in Breves-PA, in Brazil. We report here on our observations made on three different fruits which are typical examples and representative of the mesocarp microstructure as seen in more than 40 fruits which we used for *in situ* mechanical testing^33^.

### Cellular level: structural characterization

The observation of cells in higher resolution was made by scanning electron microscopy (SEM). Fractured surfaces of one fruit were gold coated and observed in a FEI Inspect S 50 scanning electron microscope. Additionally, latitudinal sections of mesocarp were embedded in polyester resin, ground with sandpaper (grit number 120, 320, 600 and 1200) and polished with alumina suspension of 1 µm and also observed in the same microscope.

SEM microscopy of the same fruit also allowed the visualization of cell walls in the characterization of the fibrillary hierarchical level. To allow microtome cutting of thin sheets, a piece of mesocarp was softened in a 10% ethylene diamine aqueous solution at room temperature for 15 days. The specimens were, subsequently, washed in 100% ethanol and dried at the critical point of carbon dioxide (5 °C and 50 bar) in a Leica EM CPDO30 equipment (Vienna, Austria) to avoid cell deformation. Finally, the specimens were covered with thin layers of gold and platinum and observed in a FEI Quanta 25 under high-vacuum mode and a FEI Magellan 400 L, which allows nanoscale resolution (FEI, Milpitas, USA).

Microtomography (microCT) was used to visualize the three-dimensional (3D) organization of cells, vascular bundles, and voids from two different fruits. This analysis was performed in a lab-based microcomputed tomograph phoenix nanotom m180 kV/20 W, tungsten target material from GE Measurement & Control (Billerica, EUA) and in a high-resolution microtomography SkyScan model 1172 100 kV/20 W tungsten target material from Bruker (Kontich, Belgium). In both types of equipment, no filter was used and the final images have a spatial resolution of 10 µm.

To estimate the amount of voids, the porosity of one mesocarp fruit without previous treatment was evaluated in volume reconstructions from µCT data achieved with a spatial resolution of 33 µm in the phoenix nanotom microCT. The images were analyzed with Fiji – ImageJ 2.0^38^ and the porosity was estimated from several volumes of interest inside the mesocarp structure. Each volume of interest consisted of a parallelepiped with a square cross-section with an edge length of 0.66 cm and a height of 250 slices. The images were first normalized with a saturated value of 0.4% and filtered with a median 3D (radius 2) filter and a bandpass filter from 3 to 40 pixels. The images were then segmented with a user-defined threshold determined to distinguish the voids from the mesocarp cells. The porosity was then calculated as follows using the volume fraction function of BoneJ plugin^39^.

1 $$porosity\,( \% )=\frac{{\rm{volume}}\,{\rm{of}}\,{\rm{channels}}\,{\rm{and}}\,{\rm{cracks}}}{{\rm{total}}\,{\rm{valume}}}\cdot 100$$

### Cellular level: fiber preferential orientation

To investigate the fiber orientation in the mesocarp and possible mesocarp layers, sections were investigated by light microscopy. A whole mesocarp was cut into two halves perpendicular to the peduncle-opercular opening line with a band saw. One of the cut surfaces was ground with sandpaper (grit numbers 800, 1200, 2400 and 4000) and observed with an digital microscope (VHX 100: Keyence Deutschland GmbH).

Further, another whole mesocarp was cut into pieces with a band saw, and the resulting blocks were embedded in polyester resin, ground with sandpaper of decreasing roughness (grit numbers 120, 320, 600 and 1200) and polished with alumina suspension of 1 μm. An Olympus BX41M-LED optical microscope was used to investigate the fiber orientation in greater detail. The polished surfaces were prepared in different directions to observe the mesocarp thickness parallel and perpendicular to the peduncle-opercular opening line.

### Fibrillar level: microfibril angle

To measure the distribution of the microfibril angle f(µ) of fibers in a bundle, small-angle X-ray scattering (SAXS) was used. One mesocarp piece from was sectioned in a Rotary Microtome HM 355 S from MICROM International in slices with a thickness of 10 to 30 µm. Subsequently, one bundle with several fibers from the innermost region of the mesocarp was isolated from the sclereids cells with two pointed metallic tools in a stereo microscope Olympus SZ61. This was only possible in the innermost region because the bundles there are more oriented and less entangled than in the other mesocarp regions. The fiber bundle was aligned using adhesive taping, which was placed in the sample holder of the Bruker Nanostar SAXS instrument. The X-ray radiation employed was generated from a Cu sealed tube fine-focus X-ray source (K_α_ = 1.54184 Å with a potential of 40 kV and a current of 35 mA).

The distance between the detector and sample is 106.4 cm, in an evacuated chamber. A scan of 6000 seconds was performed, followed by a standard scan of 3000 seconds, using glassy carbon between sample and detector. The subsequent blank scan of the adhesive tape followed the same procedure and its transmission factors were subtracted from sample data. Area integration reduced the two-dimensional data to a one-dimensional curve. The intensity of the scattered radiation was integrated over *q (q* = 4πsinθ/λ) and plotted versus the azimuthal angle χ^40^. As the fiber cells in the mesocarp have a circular cross-section an approach described by Fratzl *et al*.^41^ and also adopted by Lichtenegger *et al*.^40^ to evaluate cells with round cross-sections were used. After integration, the experimental data were fitted, where k, m, l, and n are the fit parameters:

2 $$S=k.{co}{{s}}^{{m}}\chi +l.{co}{{s}}^{{n}}\chi $$

According to Fratzl *et al*.^41^, the microfibril angle distribution f(µ) may be expressed as follows:

3 $$f(\mu )=\frac{k.(m+1).{u}_{m}.{co}{{s}}^{{m}}\chi +l.(n+1).{u}_{n}.{co}{{s}}^{{n}}\chi }{k.{u}_{m}+l.{u}_{n}}$$

with

4 $${u}_{m}={\Gamma }\left(\frac{m+2}{2}\right)/{\Gamma }\left(\frac{m+3}{2}\right)$$

and Γ Gamma-function

Finally, f(µ) was fitted with a Gaussian distribution to obtain the mean microfibril angle and a distribution width. The distribution f(µ) describes the probability of the microfibril pointing in a certain direction and tilted by an angle µ with respect to the longitudinal axis of the cell. The function f(µ) would be a constant equal to 1 for a cell with microfibrils in a totally random orientation. Accordingly, f(µ) with a peak at µ = 0 indicates that the microfibrils are preferentially oriented along the longitudinal fiber axis^41^.

## Data Availability

The data that support the findings of this study are available from the corresponding author, M.S., upon request.
